# Field data on sea ice restoration by artificial flooding in subarctic Canada

**DOI:** 10.1016/j.dib.2025.112147

**Published:** 2025-10-08

**Authors:** Cody C. Owen, Soroosh Afzali, Willem Schellingerhout, Tom Meijeraan, Fonger Ypma

**Affiliations:** Arctic Reflections, Paardenmarkt 1, 2611 PA, Delft, Netherlands

**Keywords:** Snow, Albedo, Snow ice, Melting, Desalination processes, Insolation, Ablation, Earth cooling approach

## Abstract

A field campaign in the Milan Arm of Pistolet Bay in Newfoundland, Canada was conducted to gather data on sea ice restoration by artificial flooding between February and May of 2025. Sea ice thickening was initiated by pumping sea water from below the first-year sea ice onto the surface without significantly modifying the overall snow cover beforehand. Pumping consisted of 84 discrete events, for which GPS location, pumping start time and duration, and local snow and ice thicknesses were recorded. Remote data collection and monitoring were executed by three thermistor chains, three radiation sensors, and one anemometer. All remote measurement systems remained in the field until recovery of the floating systems following ice breakup in late spring. Additionally, coring systems were used to extract 10 ice cores for analysis of temperature and bulk salinity profiles through the ice depth to assess the effect of artificial flooding on sea ice formation and ablation. Two of the ice cores were used and three seawater samples were collected for analysis of the biological content of phytoplankton. Transects of surface composition across select flooded sites were assessed for the formation and solidification of ice and slush layers. Snow thickness and density data were sampled for a representative region of the entire site to assess spatial variability. All these data were complemented by timelapse camera imaging from each monitoring station and aerial drone imaging, including thermal imaging, of the entire region.

The dataset can be used to investigate the physical processes involved in sea ice growth before, during, and after flooding. The dataset can be used, in a limited manner, to understand the formation, growth, and ablation of snow ice. The radiation data can be used to analyze the surface radiation fluxes of the parent, flooded, and melting ice. The data gathered during the melting season can be used to investigate the melting of thickened sea ice in comparison to that of natural sea ice. The data on bulk salinity can be used to investigate short-term brine migration. The data on phytoplankton content can be used to assess its change due to the impact of flooding. Combining the various data, thermodynamic ice growth and melt models of sea ice, including snow, slush, and snow ice, can be validated. The understanding of rain and meltwater drainage events could be improved and flow models for simulation of artificial flooding of snow-covered first-year sea ice could be further developed using the data. Aerial imagery obtained by drone provides insights into the flooding behavior of water over snow-covered ice, allowing for the detection and temporal tracking of both visibly impacted and visually concealed areas that may not be apparent to the naked eye.

Specifications Table

**Table:** 

Subject	Earth & Environmental Sciences
Specific subject area	Artificial flooding of snow-covered first-year sea ice and associated geophysical freezing and melting processes.
Type of data	Raw (.csv, .xlsx), Images (.jpg), Video (.mp4).
Data collection	Field data were collected between 18 February and 6 May of 2025 (77 days) in the Milan Arm of Pistolet Bay in Newfoundland, Canada. The test sites were flooded between 27 February and 9 March. The measurement stations collected data remotely between 22 February and 6 May. 10 ice cores and 3 water samples were extracted between 1 March and 12 March. The data collection involved various instruments. Thermistor chains (Snow and Ice Mass Balance Apparatus (SIMBA) – SAMS Enterprise) were installed in the ice cover and used to remotely monitor temperature, with one installed at each site. Radiometers (Apogee Instruments SN-500 Net Radiometer – METER Group), consisting of upward- and downward-looking pyranometers (SP-510-SS and SP-610-SS) and pyrgeometers (SL-510-SS and SL-610-SS), were installed on the measurement station at each site to measure radiative fluxes. Wind speed and direction were measured using an ultrasonic anemometer (ATMOS 22 Ultrasonic Anemometer – METER Group) installed on the measurement station at Site B. The radiometers and anemometer were connected to a ZL6 Advanced Cloud Data Logger (METER Group) at each measurement station for remote data collection and access during the flooding, freezing, and melting periods. The data loggers recorded temperature and barometric pressure. Ice thickness measurements were performed with the Kovacs Mark II coring system (Kovacs Enterprise) to extract and measure ice cores and with the Kovacs ice thickness kit (Kovacs Enterprise) consisting of an ice auger and ice thickness gauge. The water depth was measured with the ice thickness gauge. The snow depths were measured with a folding ruler. The snow density was measured with a Backcountry Snow Pit Kit (Snowmetrics). Flooding tests were conducted using the custom-made EMV-690 flood pump (Erikssons Mekaniska Verkstad AB) for controlled water discharge. For each flooding event, a borehole was drilled with an auger and the pump was started, inserted into the borehole, and the
	duration was recorded. In situ ice coring was performed using a Kovacs Mark II coring system (Kovacs Enterprise) to extract ice samples for analysis. Ice cores were used to analyze temperature in situ, by drilling pilot holes in the ice core on site with an electric drill, and the temperature was measured with a TFX 410 core thermometer with a fixed Pt 1000 Probe (Xylem Analytics Germany Sales GmbH & C. KG). Bulk salinity of each ice core was measured by melting the ice samples and using a GMH 3431 Conductivity Meter (Senseca Germany GmbH). Water samples were collected with lidded plastic containers. The biological samples were preserved with 0.04 % Lugol’s acid or alkaline solution. Field activities were documented with Tikee 3 PRO+ (on measurement station at Site B) and Tikee 2 PRO+ (on measurement stations at Site A and Site C) cameras (Two Sony Exmor R 16 Mpx image sensors). RGB-color and thermal aerial imagery of the flooded sites and reference ice were captured using a DJI Mavic 3T (DJI Sky City) drone before, during, and several days after the flooding and subsequent freezing events. The drone was also used to document procedural activities throughout the data collection period. The data provided are raw data. No data have been excluded.
Data source location	Milan Arm, Pistolet Bay, Newfoundland, Canada. All activities were conducted between 51.48° and 51.50° latitude (positive is north) and between −55.72° and −55.67° longitude (negative is west).
Data accessibility	Repository name: Zenodo Data identification number: https://doi.org/10.5281/zenodo.16686796 Direct URL to data: https://zenodo.org/records/16686796
Related research article	*None.*

## 1 Value of the Data

• These data enable the study of the effect of flooding snow-covered first-year sea ice on snow ice formation, congelation ice growth, and melting processes of the ice cover in the subarctic. The data may also be used to assess the impact of artificial surface flooding of sea ice on sea ice biology in the subarctic.
• Thermodynamic models of the growth and decay of snow-covered first-year sea ice in the subarctic with snow ice and slush layers can be validated with the data.
• The short- and longwave radiative fluxes from the surface during the flooding, thickening, and melting of snow-covered first-year sea in the subarctic ice may be investigated, especially in terms of sea ice thickening as a sea ice restoration approach.
• The data may be applied as a reference dataset for further analysis of variation in ice properties, such as the proportion of snow and congelation ice over time, due to climate change (e.g. more frequent sea ice flooding from rain in the subarctic).
• Policymakers, engineers, researchers, and the social and public sector leaders may benefit from this dataset by informing their decisions on the use of sea ice restoration for, e.g., solar radiation management or climate adaptation methods in the subarctic.
• The data can be used to evaluate and refine the techniques and parameters—such as pump type, pump flowrate, distribution methods of flooding and spraying, and snow management via plowing or channeling—involved in artificially thickening sea ice through surface flooding.

## 2 Background

Concurrent with global decarbonization, efforts to address anthropogenic climate change include assessment of a solar radiation management (SRM) approach wherein sea ice is thickened in winter via artificial surface flooding with seawater. Multiple theoretical studies have shown the potential for Arctic sea ice preservation, despite global warming, by flooding [[1], [2], [3]]. Without Arctic ice management, other significant SRM would be needed to prevent the loss of summer Arctic sea ice while greenhouse gas emissions continue to increase [4].

While natural surface flooding of sea ice has been well studied [[5], [6], [7], [8], [9], [10], [11], [12]], its relevance to artificial flooding is uncertain, which has motivated field experiments, conducted in the Vallunden lagoon in Svalbard in 2024, which gathered data on the physical processes associated with artificial flooding of snow-covered Arctic sea ice [13]. The current work follows similar methods and presents field experiments designed to gather data on the physical processes involved during the artificial flooding of snow-covered subarctic sea ice. The data collected may contribute to the assessment of sea ice restoration via flooding, and may also be useful in understanding the effect of natural flooding events from rain during winter on snow and ice cover.

## 3 Data Description

**Fig. 1:**
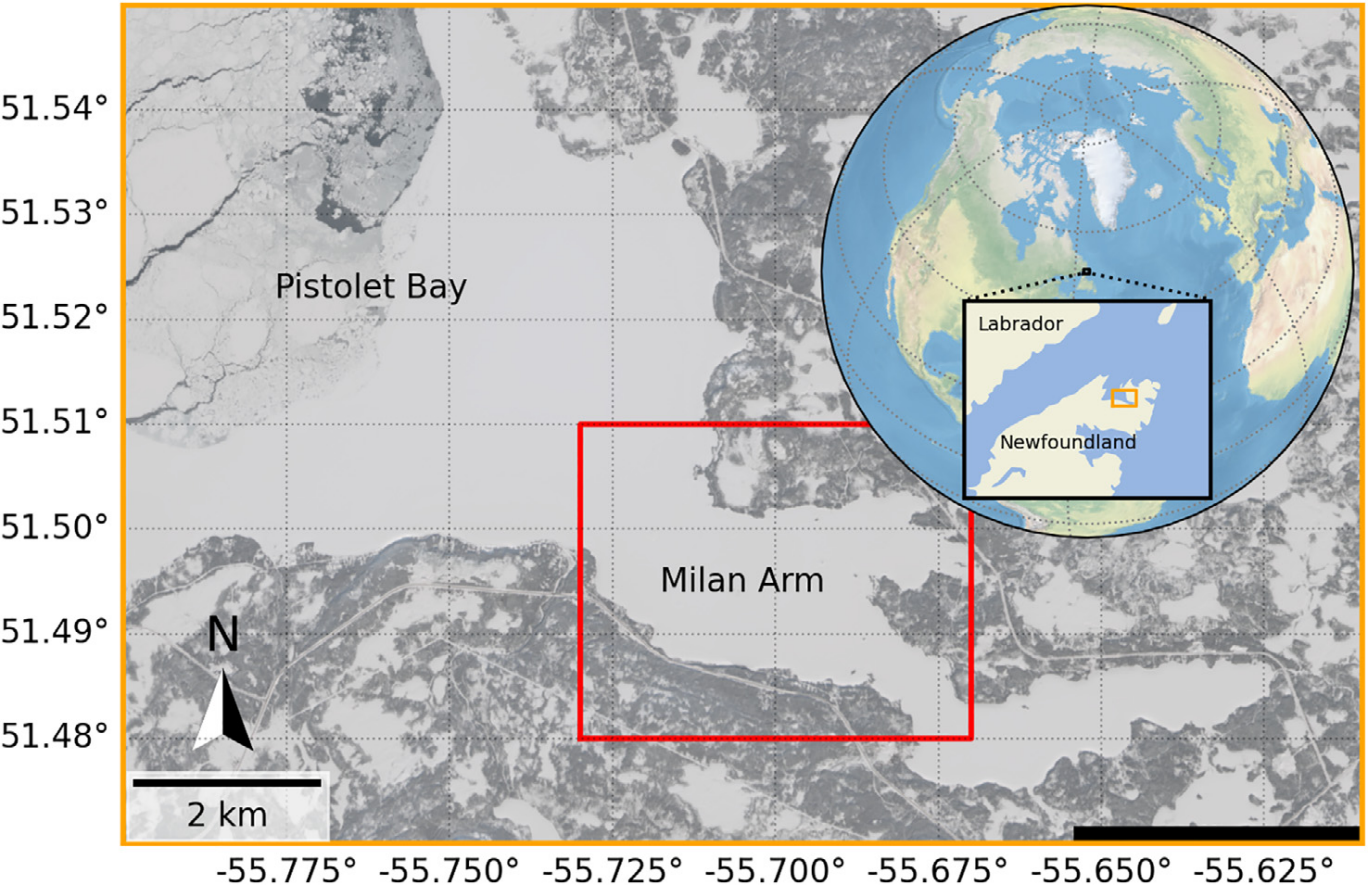
Sentinel-2 L2A satellite image of the field campaign location in the Milan Arm on 4 March 2025 from the Copernicus Data Space Ecosystem Browser. Inset depicts the field campaign location in Pistolet Bay in northern Newfoundland, Canada.

The data were collected in the Milan Arm of Pistolet Bay in northern Newfoundland, Canada between 18 February and 6 May of 2025 (77 days) (see Fig. 1) [14]. The data description is divided into four sections. The first section describes the data collected by the measurement stations which were installed into the ice cover. The second section describes the conditions of the region, including snow depth, ice thickness, water depth, and their GPS coordinates, among other data. The third section describes the data gathered by coring ice samples. The fourth section describes the images taken by the timelapse cameras and aerial drone.

### 3.1 Measurement stations

**Fig. 2:**
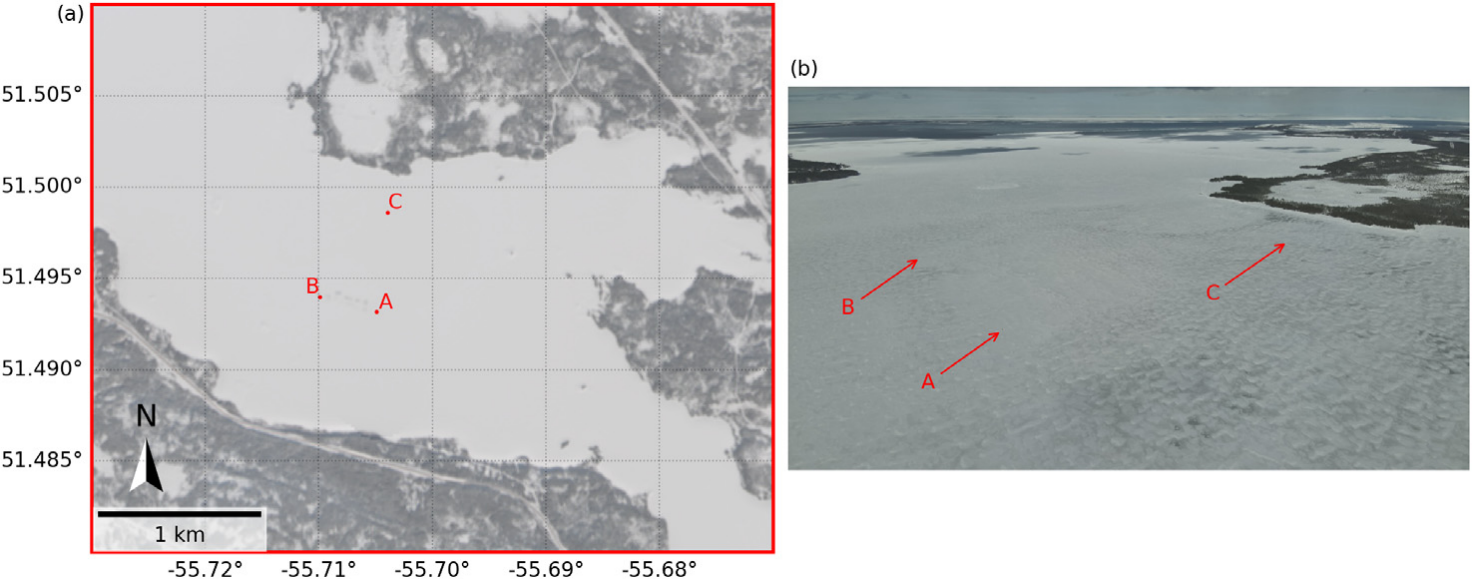
Measurement station sites: (a) Sentinel-2 L2A image of the measurement station Sites A, B, and C at the field campaign location in Milan Arm on 4 March; and (b) aerial drone photograph of the measurement station sites on 9 March. Note that Sites A and B were flooded sites, and Site C was an unaltered reference site.

Three measurement stations (Sites A, B, and C) were installed in the ice cover for remote monitoring of the snow, ice, water, and meteorological conditions (see Fig. 2). A summary of the names, coordinates, and measurement systems for each measurement station is provided in Table 1.

**Table 1:** Description of the three measurement stations.

Name	GPS coordinates (latitude, longitude) [°]	Measurement systems	Measurement period
Site A	51.4931623, −55.7049045	SIMBA: thermistor chain	22–02–2025 to 06–05–2025
		Data logger: radiometer	28–02–2025 to 06–05–2025
		Tikee: timelapse camera	28–02–2025 to 10–03–2025
Site B	51.4939815, −55.7099075	SIMBA: thermistor chain	24–02–2025 to 06–05–2025
		Data logger: radiometer, anemometer	28–02–2025 to 06–05–2025
		Tikee: timelapse camera	28–02–2025 to 10–03–2025; 11–03–2025 to 30–04–2025
Site C	51.4986209, −55.7039219	SIMBA: thermistor chain	24–02–2025 to 06–05–2025
		Data logger: radiometer	28–02–2025 to 06–05–2025
		Tikee: timelapse camera	04–03–2025 to 10–03–2025

The thermistor chain measured the vertical temperature profile along a total length of approximately 380 cm with a spatial resolution of 2 cm. The sampling rate was set to 2 h from 22–02–2025 to 25–02–2025 and to set 3 h from 25–02–2025 to 06–05–2025. The thermistor chain had a special air temperature sensor which was unaffected by solar radiation. Additionally, the thermistor chain performed heating cycles every 24 h, during which the thermistor chain was heated by resistors and the relative temperature change was recorded at two moments during the heating. This heating was performed to assist with identifying boundaries between media surrounding the thermistor chain (e.g. air, snow, ice, water) by highlighting differences in the rate of heat transfer along the chain.

The radiometer measured the incident and reflected shortwave radiation with a pyranometer, and the incoming and outgoing longwave radiation with a pyrgeometer. The anemometer at Site B measured wind and gust speed, and wind direction. The radiometers and anemometer had a sampling rate of 15 min during the period of 28–02–2025 to 06–05–2025.

The data from the thermistor chains, and GPS data from the SIMBA control boxes, are provided in a separate ‘.csv’ file for each measurement station site (Sites A,^1^ B,^2^ and C^3^), which can be found in the data repository [14]. Data from the radiometers and anemometer are provided in a separate ‘.csv’ file for each measurement station (Sites A,^4^ B,^5^ and C^6^), which can also be found in the data repository. The content of each of these files is explained in the accompanying ‘README.pdf’ file in the data repository.

**Fig. 3:**
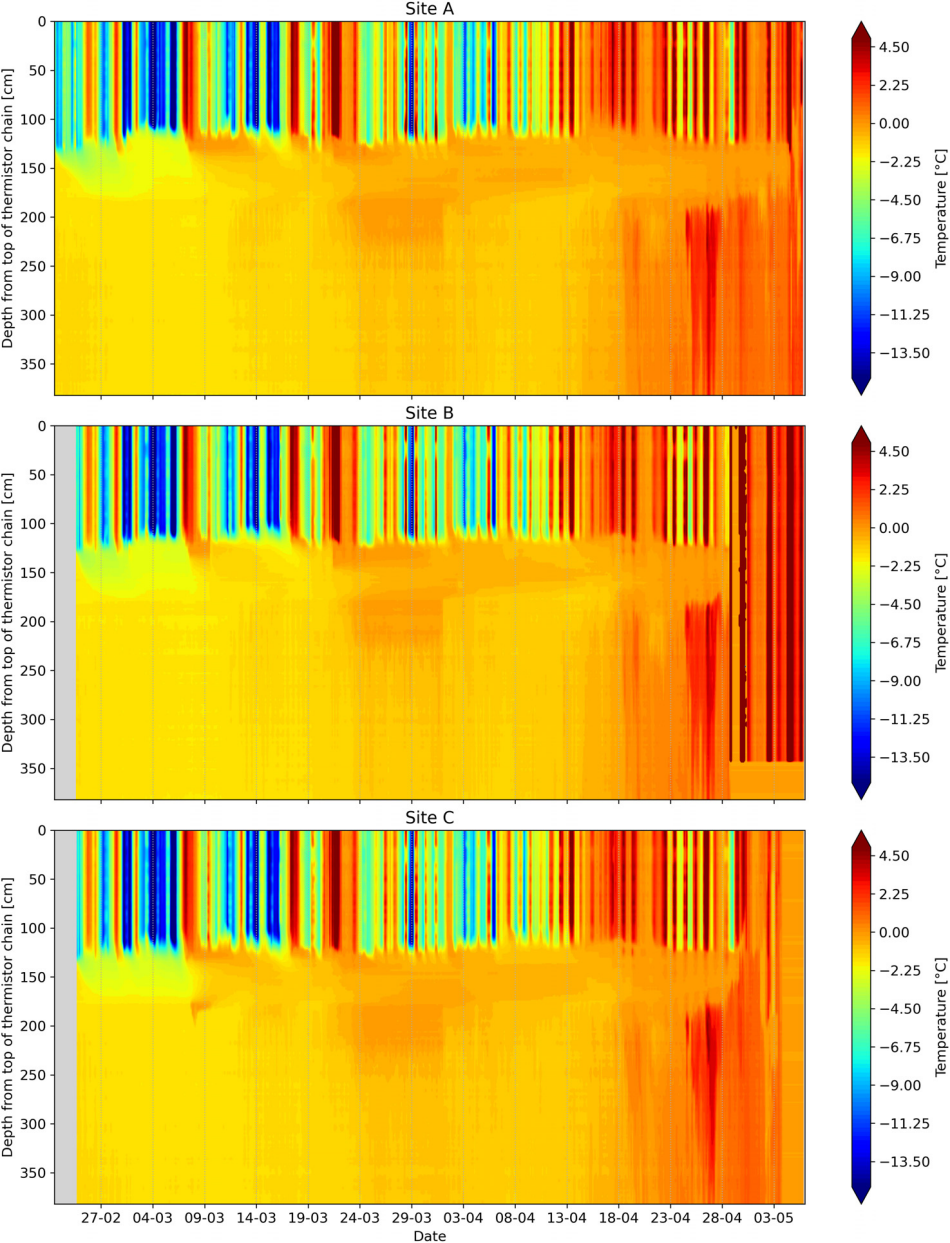
Example visualization of vertical temperature profiles from the thermistor chains at Sites A, B, and C.

**Fig. 4:**
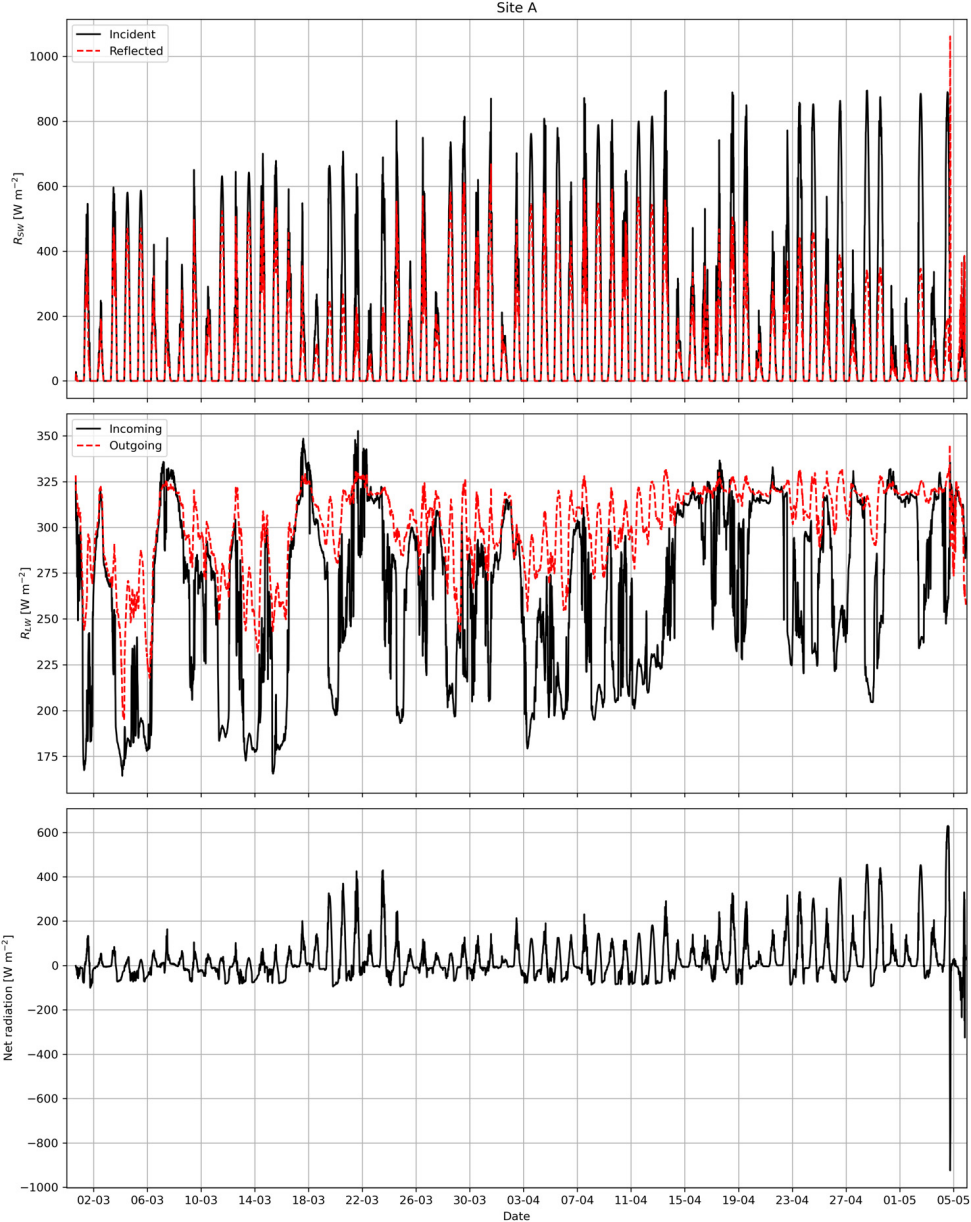
Example visualization of incident and reflected shortwave radiation (R_SW_), incoming and outgoing longwave radiation (R_LW_), and the net radiation from the radiometer at Site A.

**Fig. 5:**
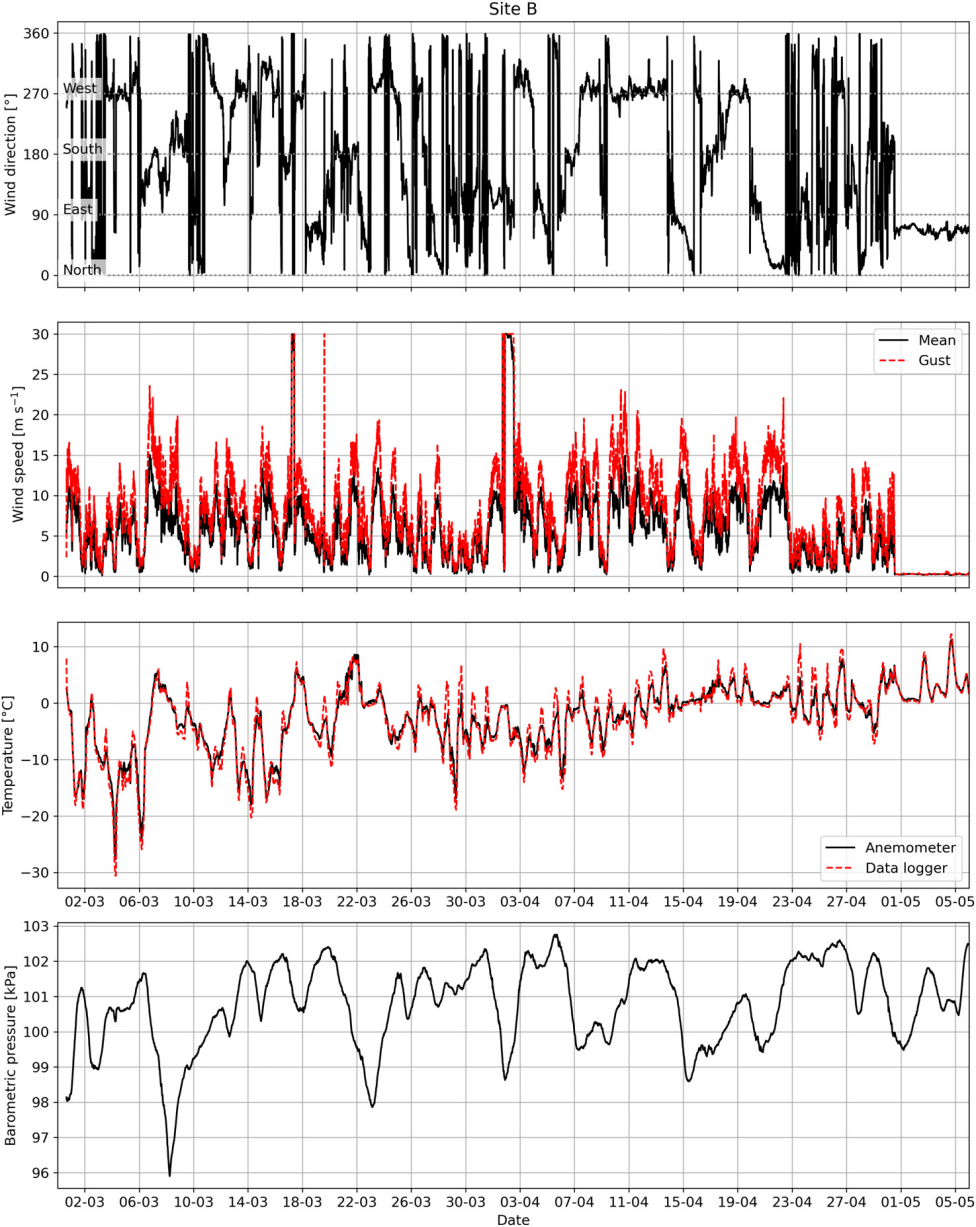
Example visualization of meteorological data at Site B.

An example visualization of the data collected by the thermistor chain for each measurement station site is shown in Fig. 3. An example visualization of the data collected by the radiometer at Site A is shown in Fig. 4. An example visualization of the data collected by the anemometer and data logger at Site B is shown in Fig. 5.

### 3.2 Region conditions

**Fig. 6:**
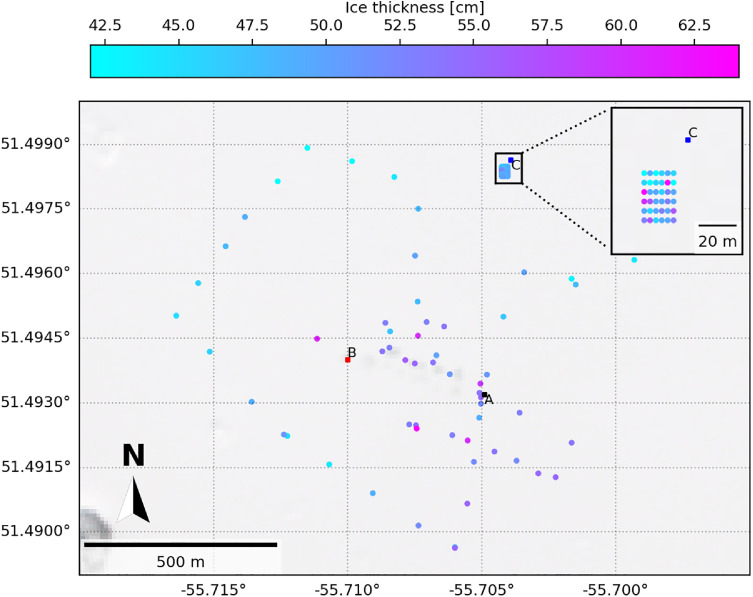
Example visualization of the map of select ice thickness measurements between 19 February and 9 March, superimposed on the Sentinel-2 L2A satellite image taken on 4 March. Sites A, B, and C are shown for reference. The inset shows the detailed, gridded snow study area.

Snow depth, ice thickness, and water depth measurements, and points of interest, and the corresponding GPS coordinates, were collected throughout the region,^7^ and are available in the data repository [14]. Additionally, for better assessment of spatial variability, snow depth, ice thickness, and select snow density measurements from a detailed, gridded area in the region^8^ are available in the data repository. A description of the region conditions data is provided in the accompanying ‘README.pdf’ file in the data repository. An example visualization of the ice thickness measurements of the region is shown in Fig. 6. The snow and water depths can be visualized in a similar manner.

### 3.3 Surface composition sampling

During the campaign, at several pumping locations, measurements were conducted to better understand the composition and layering of surface materials, including ice, slush, and snow. In some cases, additional parameters such as the salinity and temperature of the slush layer were also recorded. All collected data from these surface composition assessments^9^ are available in the data repository [14].

### 3.4 Pumping operations

**Fig. 7:**
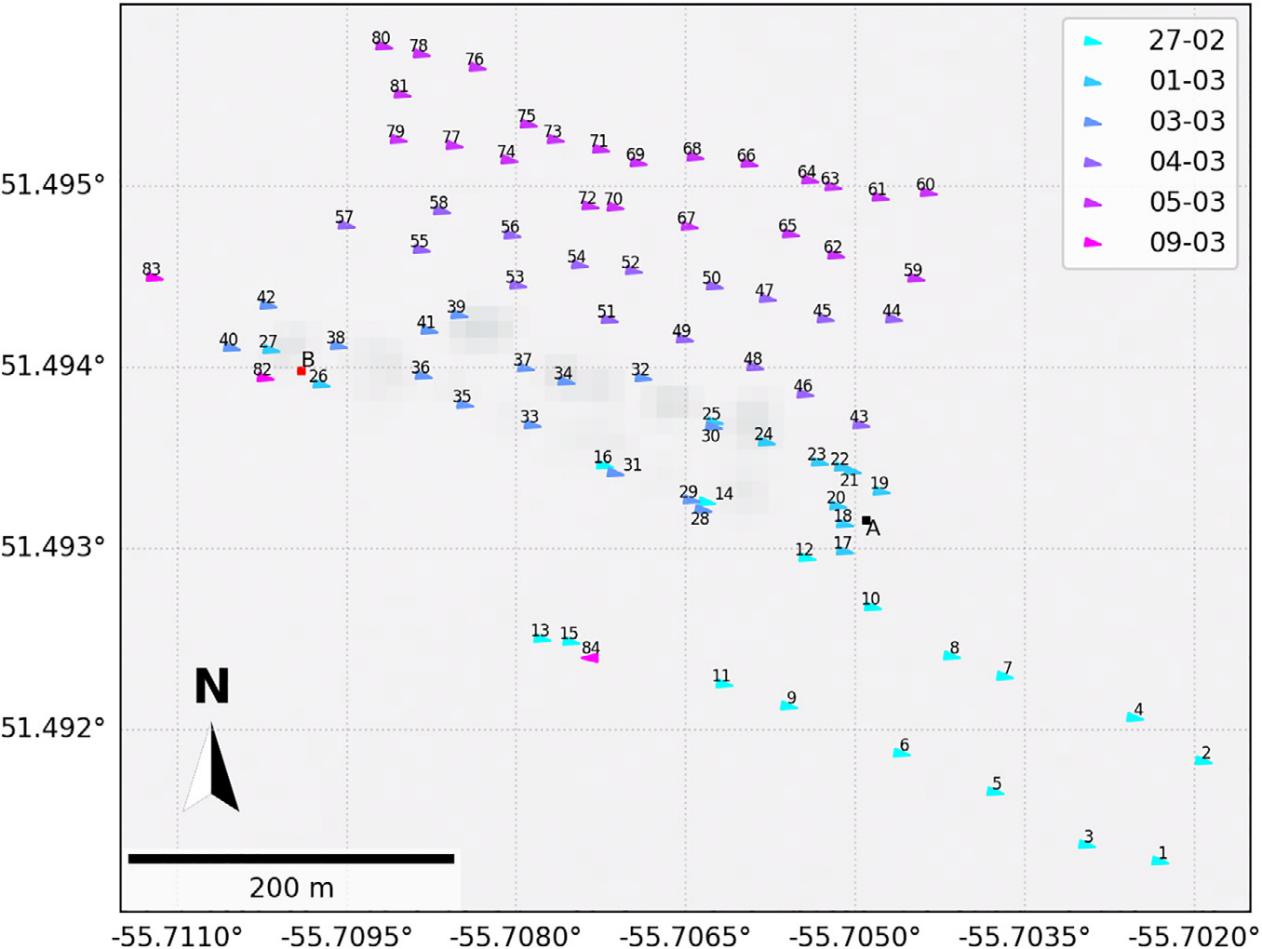
Example visualization of the map of flooding operations with site numbers, superimposed on the Sentinel-2 L2A satellite image taken on 4 March. Sites A and B are shown for reference.

The pumping operations, including GPS coordinates, start and stop times of pumping, pumping direction, and relevant meteorological data extracted from the measurement stations,^10^ are described in the data repository [14]. A description of the pumping operations data is provided in the accompanying ‘README.pdf’ file in the data repository. An example visualization of the pumping locations, directions, and dates is shown in Fig. 7.

### 3.5 Ice core sampling

**Fig. 8:**
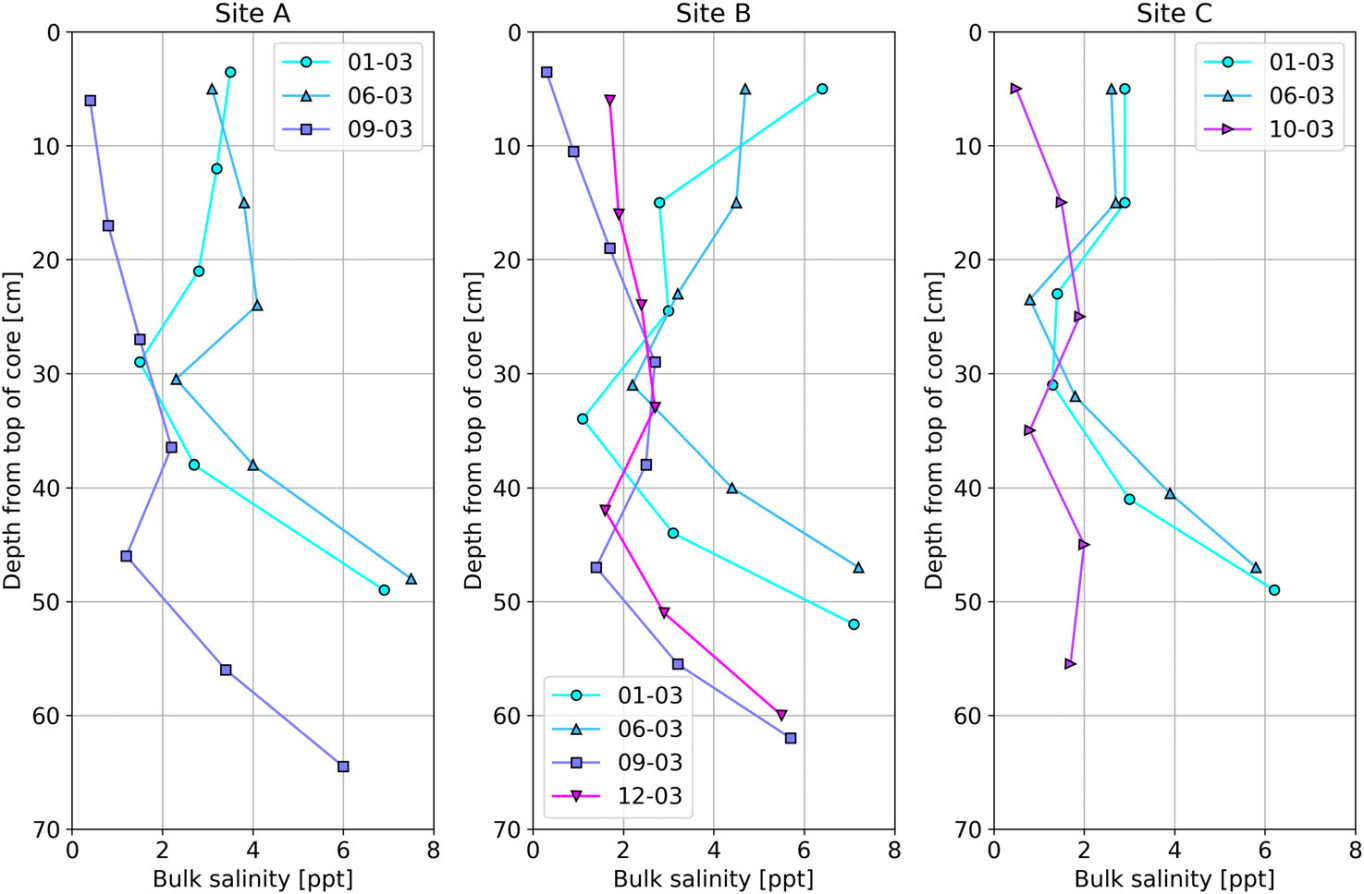
Example visualization of ice core salinity profiles for Sites A, B, and C, with the mean of the start and end depths plotted for each salinity measurement.

Ice cores were extracted within 3 m from the southern side of each measurement station at Sites A, B, and C and analyzed. A summary of all ice cores collected during the field campaign, ice core temperature and bulk salinity measurements, and their respective site conditions,^11^ are provided in the data repository [14]. A description of the ice core data is provided in the accompanying ‘README.pdf’ file in the data repository. An example visualization of the bulk salinity profiles measured for each ice core is shown in Fig. 8. The ice core temperature profiles can be visualized in a similar manner.

### 3.6 Sea ice biological sampling

Two ice cores and several water samples were collected for measurements of biological content of phytoplankton.^12^ A description of the ice core data is provided in the accompanying ‘README.pdf’ file in the data repository.

### 3.7 Imaging

#### 3.7.1 Timelapse camera

Timelapse videos from Sites A,^13^ B,^14^ and C^15^ are provided in ‘.MP4’ format, based on photographs taken with a frequency of 15 min. Photographs were taken from 28 February to 10 March at Sites A and B, and from 4 March to 10 March at Site C. The cameras were paused in the evening on 10 March to make a backup of the data from the local storage, and photographic capture was resumed for all sites from 11 March to 30 April. Note that only the timelapse camera at Site B was recovered at the end of the melting season.

#### 3.7.2 Aerial drone

**Fig. 9:**
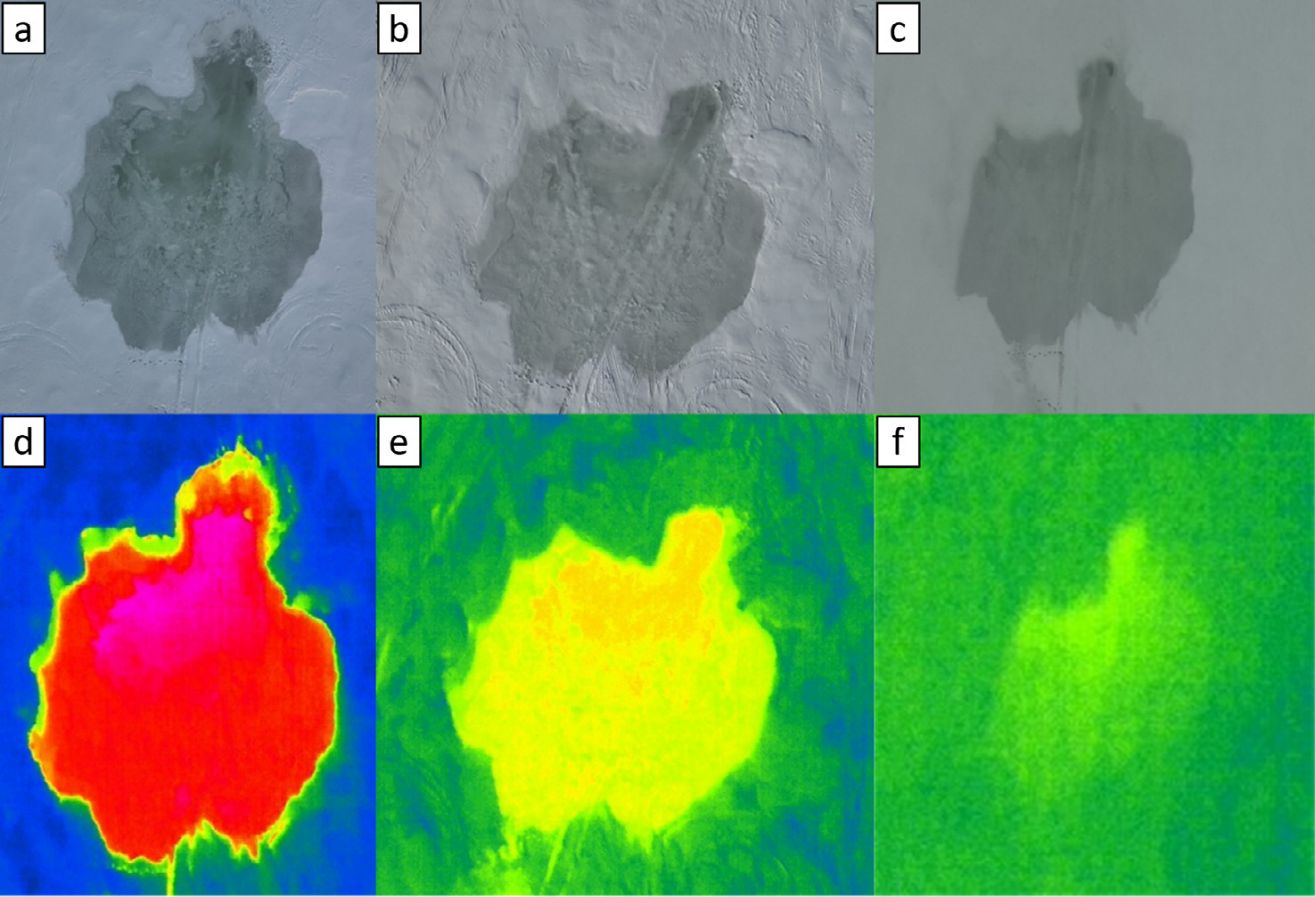
Example RGB (a)-(c) and thermal (d)-(f) aerial drone images for Site 51, wherein (a) and (d) show the flooded site on the day of pumping, (b) and (e) show the site one day later, and (c) and (f) show the site two days later. Note that the thermal image color-scale in this case is arbitrary.

The aerial drone captured RGB-color and thermal images of the flooded zones during and after flooding operations, and the images in ‘.JPG’ format for each flooded zone can be found in folder ‘Drone’ in the data repository [14]. The subfolders are named numerically according to the site number(s) listed in the aforementioned file ‘Pumping_operations.xlsx’. Examples of RGB-color and thermal drone aerial photography taken at different times after pumping water over snow covered ice are shown in Fig. 9. Corresponding metadata^16^ for the drone images can be found in the data repository. A summary drone video^17^ of the region is also available in the data repository to give visual context.

## 4 Experimental Design, Materials and Methods

The section is divided into four subsections. The first subsection describes the experimental design of the measurement stations. The second subsection explains the flooding procedure of the test sites and measurement station sites. The third subsection describes the ice core sampling and the methods for measurement of temperature, bulk salinity, and biological content. The fourth subsection explains imaging from the timelapse cameras at the measurement station sites and the aerial drone imaging, both with RGB-color and thermal vision, of the flooded sites.

### 4.1 Measurement stations

**Fig. 10:**
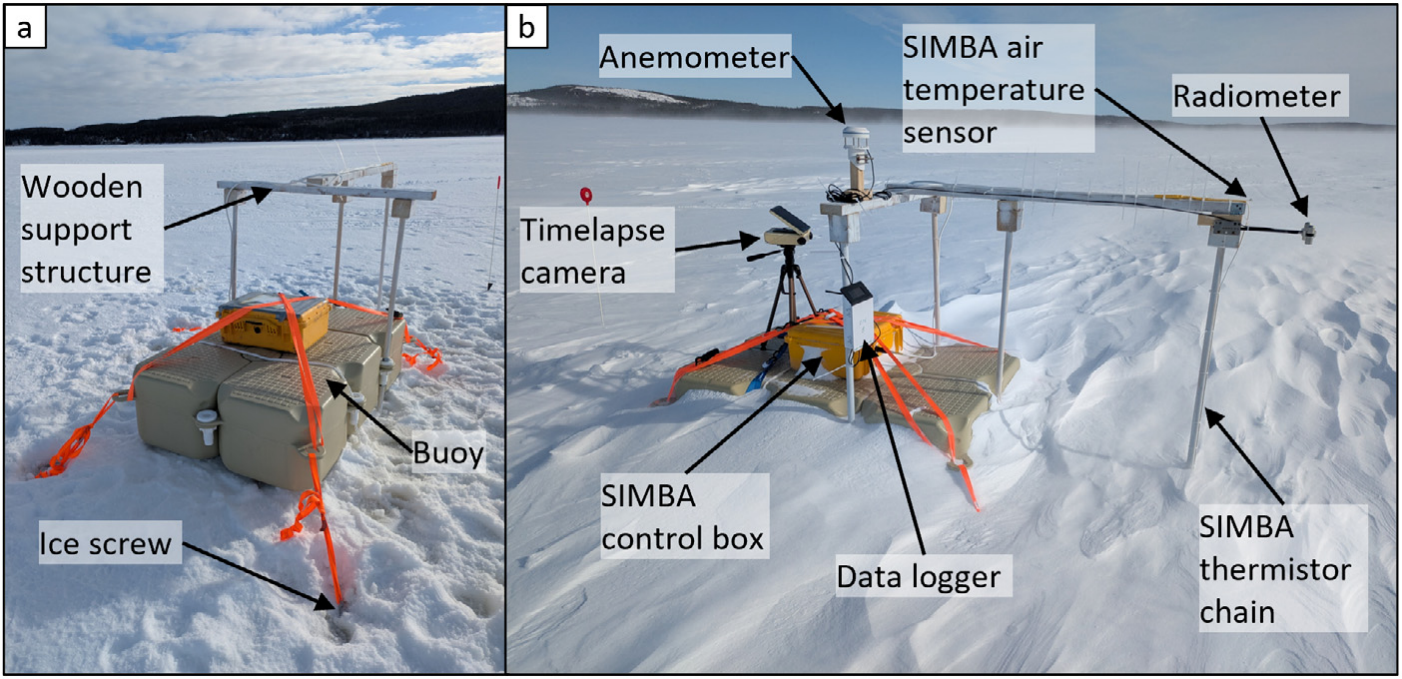
Photographs of the measurement station: (a) installation of measurement station in ice cover; and (b) deployment of all measurement instruments at Site B.

The instruments of the measurement station installed at Site B consisted of a Snow and Mass Balance Apparatus (SIMBA) control box and thermistor chain, a radiometer, an anemometer, a data logger for the radiometer and anemometer, and a timelapse camera on a tripod (see Fig. 10). The measurement station was comprised of six Sunny Dock (Dock Marine Nederland) single cubes of 50 cm by 50 cm by 40 cm with the relevant connecting pins and bolts, a wooden support structure for the sensors, and an anchoring system. The wooden support structure consisted of four legs and a crossbeam, in which three legs were clamped to the buoy and one leg was cantilevered. The anchoring system consisted of a small grapnel anchor and ice screws: the anchor was designed to slow or arrest the movement of the measurement station once the ice completely melted, and the ice screws were designed to maintain the position of the buoy relative to the thermistor chain during flooding and ice melting to prevent the thermistor chain from breaking. The buoy and support structure were designed to keep the sensors afloat and operational during the melting period, including ice-free conditions. The SIMBA control box had an Iridium satellite modem for GPS tracking for location monitoring and recovery. The thermistor chain, radiometer, anemometer, and data logger were fastened to and supported by the wooden support structure. The entire measurement station weighed about 60 kg, allowing for ease of retrieval by boat in late spring.

For installation of the measurement station, a borehole was drilled in the ice cover at the site and a small grapnel anchor was lowered through the borehole to the seabed and attached to the buoy with a rope. The buoy was placed over the borehole and oriented with the wooden support structure pointed due south. Four 21-cm ice screws (PETZL – Laser) were installed near the corners of the buoy in the ice cover and the corners of the buoy were fastened tightly to the ice screws with ratchet straps. A borehole was then drilled in the ice cover near the far end leg of the wooden support structure, in line with the leg. A ballast was attached to the end of the thermistor chain and the ballast and chain were lowered through the borehole. The thermistor chain was fastened to the wooden support structure with white cable ties, ensuring that the cable ties did not cover any of the thermistors along the chain. The snow was cleared from around the thermistor chain to accelerate the freezing of the chain in the borehole. The SIMBA control box was fastened to the top center of the buoy with ratchet straps. The radiometer was screwed to the south end of the wooden support structure with a mounting bracket, and was approximately 120 cm from the ice surface. The anemometer was mounted, approximately 155 cm from the ice surface, to a small wooden beam at the top of the support structure using its U-clamp. The data logger and the cables from the radiometer and anemometer were fastened to the support structure with cable ties. The tripod of the timelapse camera was fastened to the top of the buoy with a ratchet strap.

The measurement stations at Sites A and C followed the same experimental design and installation, with the exception that no anemometers were installed at either Site A or Site C, i.e. the anemometer was only installed at Site B.

### 4.2 Region conditions measurements

The regions conditions measurements consisted of snow depth, ice thickness, water depth, and information about those measurement locations and other points of interest in the region. Snow depth was measured using a folding ruler by pressing the ruler into the snow about 5–7 times to estimate the snow depth. Ice thickness was then measured by clearing the measurement zone of snow and using a KOVACS Mark-II coring system with electric Makita drill or with the KOVACS ice thickness kit, consisting of a 2-inch (5 cm) ice auger with electric drill and ice thickness measurement gauge. The ice thickness measurement gauge was then used to estimate the water depth.

### 4.3 Snow variability measurements

A dedicated area near Site C was selected to measure the snow depth and density variability representative of the region. On 6 March, a 5 m by 5 m grid was marked and measurements of snow depth and ice thickness were taken every meter for a total of 36 locations. Snow density measurements were performed using a Backcountry Snow Pit Kit (Snowmetrics) with a RIP 2 Cutter 250 mL snow density wedge sampler and spring scale. Snow mass measurements were taken at nominally top, middle, and bottom locations through the snow depth at six sites diagonally within the grid. Snow mass records were performed inside the Fat Truck (see later section for explanation) to prevent wind from influencing the measurements.

### 4.4 Surface composition measurements

**Fig. 11:**
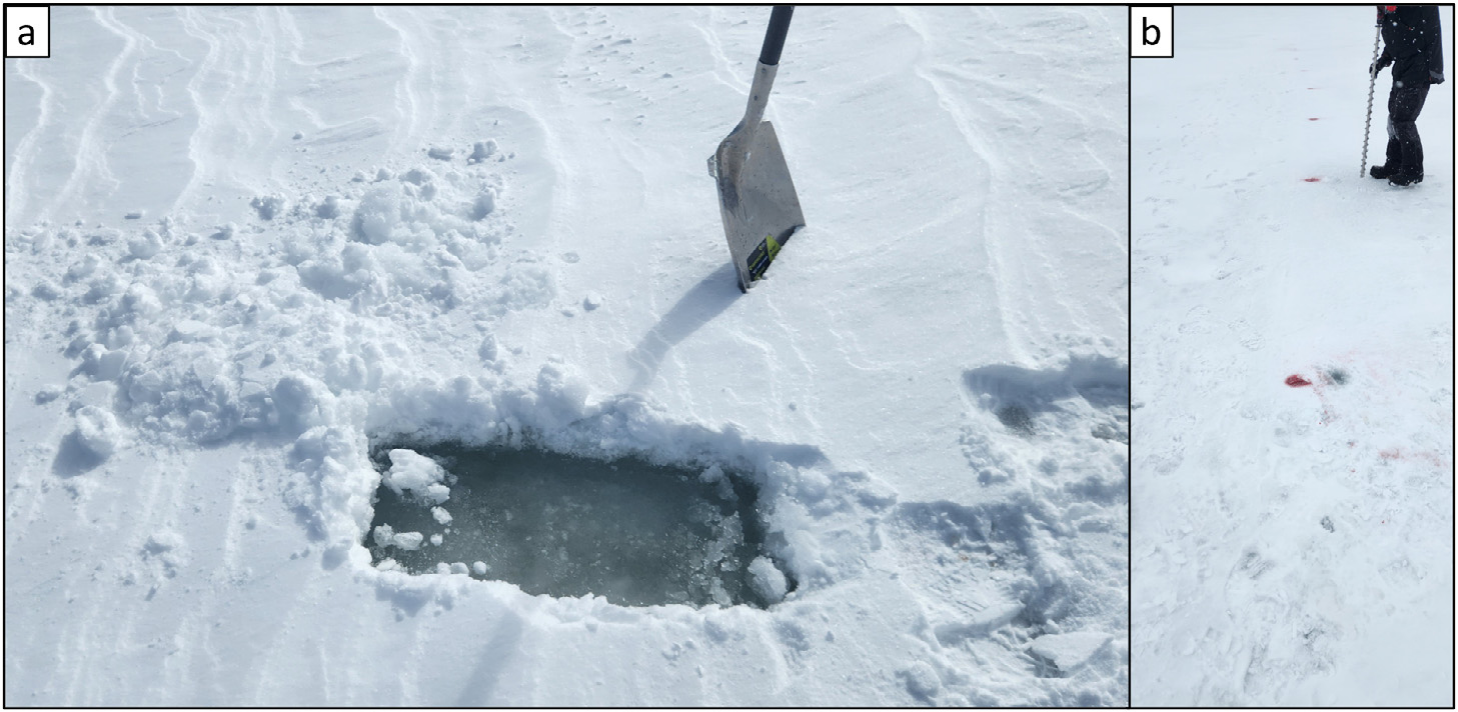
Photographs of the procedures for the surface composition sampling: (a) removal of snow and slush layers from ice surface; and (b) drilling of boreholes for ice thickness measurements.

During the campaign, at several pumping locations, measurements were conducted to better understand the composition and layering of surface materials, including ice, slush, and snow. In some cases, additional parameters such as the salinity and temperature of the slush layer were also recorded. These observations aimed to understand the composition of the surface layer over time and space, and determine how far water might distribute beneath the surface in slush form—extending beyond what is visually apparent from the top. To ensure accurate measurements, the snow and slush layers were removed prior to sampling. In situations where surface ice had formed or where total ice thickness was measured, auger holes were drilled, as illustrated in Fig. 11.

### 4.5 Flooding of test sites

**Fig. 12:**
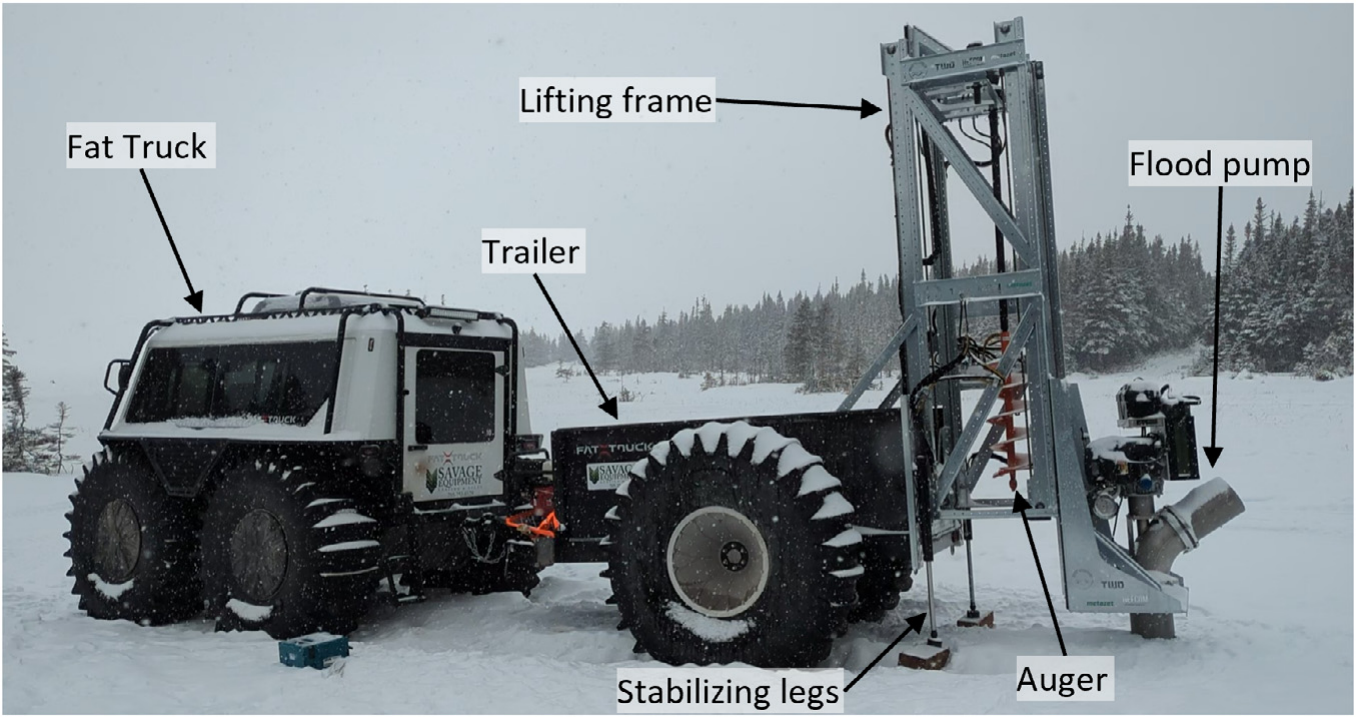
Photograph of the pumping platform.

The flooding of the snow-covered sea ice was conducted with two mobile pumping platforms, each consisting of a Fat Truck 2.8 Wagon amphibious vehicle, a Fat Truck amphibious trailer, and a custom lifting system with the EMV-690 flood pump (nominal capacity of around 300–700 m^3^ h^−1^) and a modified 12-inch (30 cm) earth auger (see Fig. 12). The lifting system consisted of a hydraulic system for skidding the lifting frame in and out of the trailer, raising and lowering stabilizing legs onto the ice cover, raising and lowering the flood pump and auger, and rotating the auger.

**Fig. 13:**
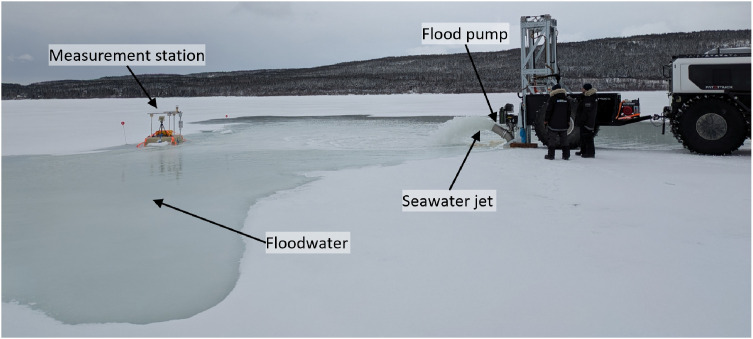
Photograph of the flooding operation at Site B on 9 March.

A flooding operation consisted of the following steps. First, the stabilizing legs were lowered, and the lifting frame was skidded out to allow the auger to drill a 30-cm borehole in the ice cover. Next, the lifting system was skidded in until the flood pump was aligned with, and then lowered into, the borehole. The flood pump was then started and a seawater jet would flood the snow cover (see Fig. 13). To prevent significant flowback of the floodwater towards the pumping platform, snow was shoveled to create a barrier between the developing pool and the pumping platform. Pumping was conducted for about 30 min or until floodwater began to surround the pumping platform, at which point the pumping was ceased and the pumping platform was moved away from the flooded site. The borehole was either plugged with a modified trash can or snow and ice to minimize drainage of the floodwater into the borehole. All trash cans were retrieved once the flooded sites had frozen. Pumping was performed downwind of the dominant wind direction to prevent water spray-induced icing on the equipment and to promote wind-driven distribution of the floodwater.

### 4.6 In situ ice coring and ice core measurements

The Kovacs Mark II ice coring system with a Makita electric drill was used to extract ice cores, with a diameter of 9 cm, from each test site within 3 m from the thermistor chain on the south side of the measurement station. Each ice core was immediately removed from the core barrel and the temperature profile was recorded along the length of the core. The same core was then prepared for bulk salinity measurements.

#### 4.6.1 Temperature profile measurements

**Fig. 14:**
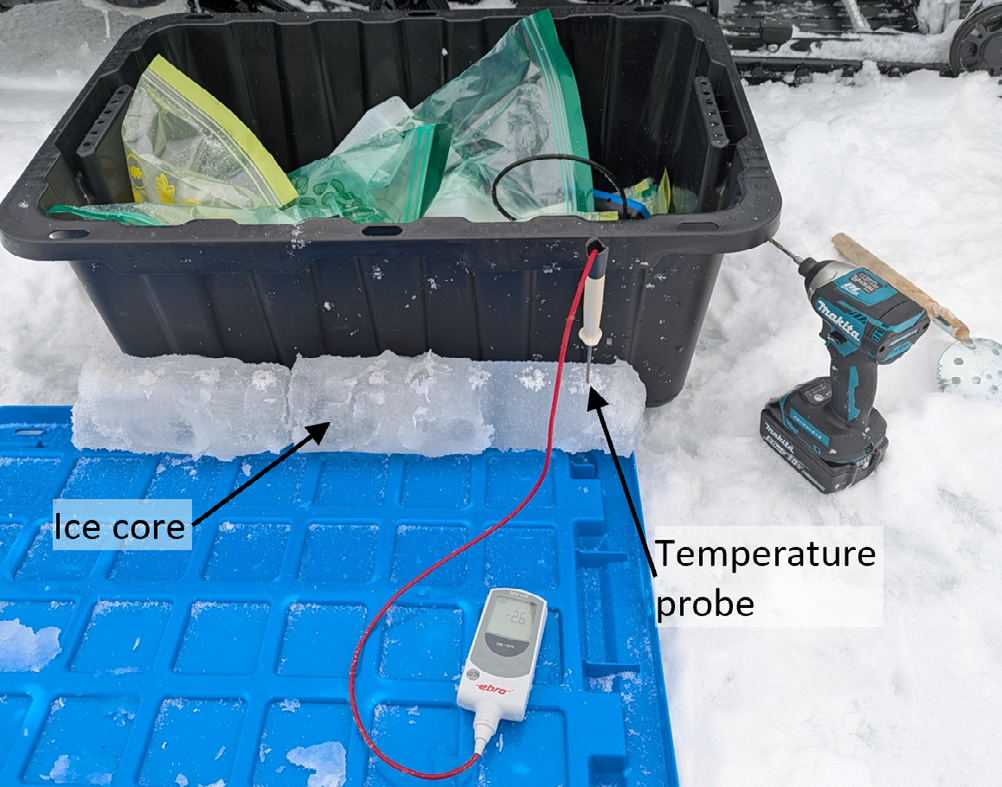
Photograph of ice core temperature profile measurements on site.

Each ice core, after being extracted from the ice cover, was immediately removed from the core barrel and a Makita electric drill with a small drill bit was used to drill holes every 5 cm along the length of the ice core to the center of the core. A temperature probe was inserted in each hole and the temperature was recorded once the temperature reached steady-state, or after about 10 s (see Fig. 14).

#### 4.6.2 Bulk salinity profile measurements

**Fig. 15:**
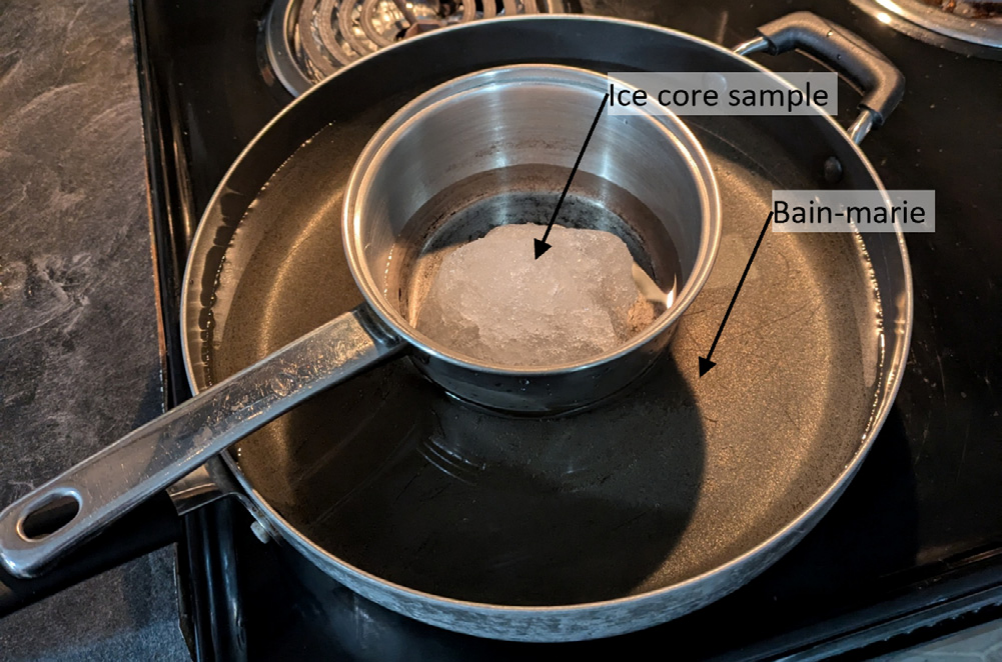
Photograph of ice core in bain-marie for salinity measurements.

Once the temperature measurements were completed, each ice core was cut with a pruning saw into sample pieces of roughly 10 cm in length (or less if there was a break in the ice core from coring) and placed inside two labeled, sealable plastic bags. The ice samples were transported to the accommodations, and each sample was removed from the bags and melted in a bain-marie (or water bath) with heating water of ≤35 °C (see Fig. 15). The meltwater was stirred until the salinity equilibrated and the salinity and temperature of the meltwater were recorded with a salinity meter. Care was taken to wash and dry the pan between melting samples.

### 4.7 Sea ice and water biological measurements

Two of the ice cores from the salinity measurements were also used for investigating biological content of phytoplankton. Moreover, water samples from surface flooding or from beneath the sea ice were collected for this purpose. The (melt-)water from each sample was transferred to a lidded plastic container and Lugol’s acid or alkaline was added to make an approximately 0.04 % solution to preserve the biological sample. Once all samples were collected, each container was further sealed with duct tape, placed in sealable bag, arranged in a box, and shipped from St. Anthony, Canada to Haren, Netherlands for analysis of the biological content of phytoplankton at Waardenburg Ecology.

### 4.8 Imaging

#### 4.8.1 Timelapse cameras

**Fig. 16:**
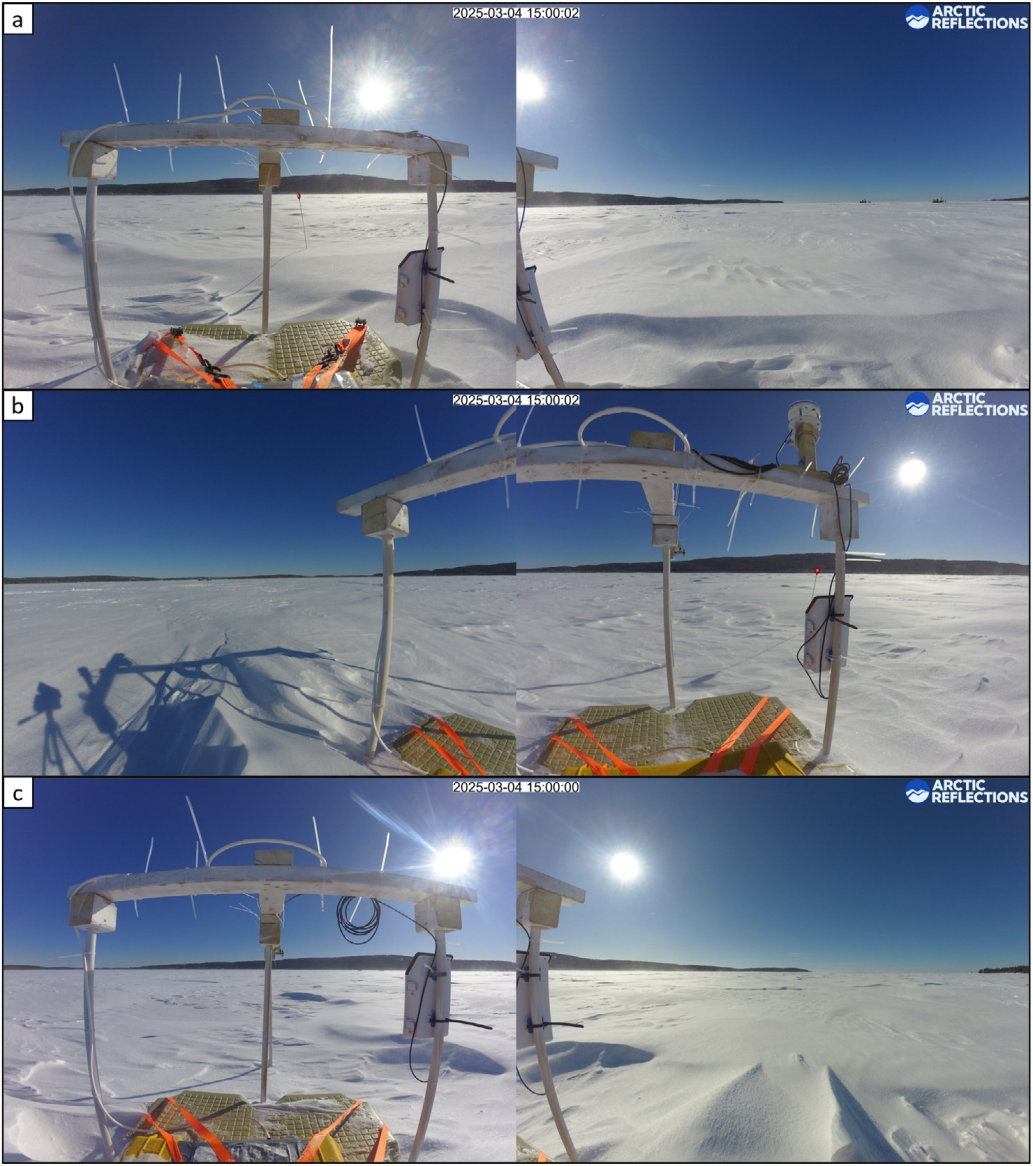
Example timelapse photographs on 4 March from (a) Site A; (b) Site B; and (c) Site C.

The timelapse cameras, consisting of two lenses, were mounted on the buoy of each respective measurement station to visually monitor the flooding and melting of the snow and ice cover around the thermistor chain (see Fig. 16). The camera lenses faced approximately south and west for Site A, south and east for Site B, and south and west for Site C.

#### 4.8.2 Aerial drone

**Fig. 17:**
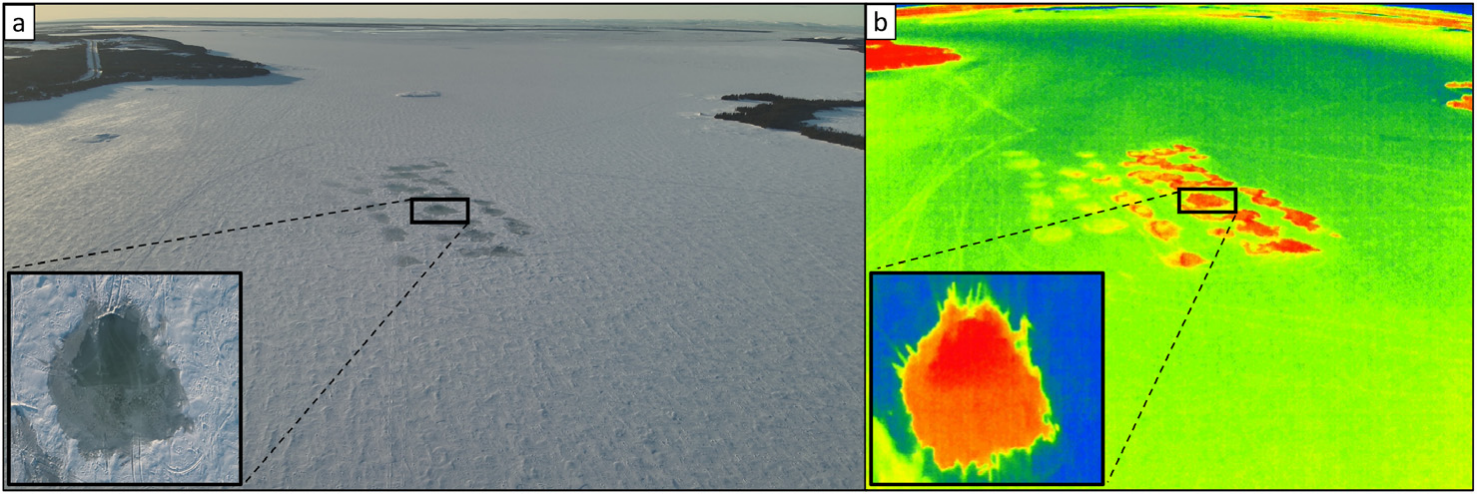
Example aerial drone images of the overall flooding operations on the evening of 5 March in (a) RGB-color; and (b) thermal. The insets show a detailed overhead view of Site 67. Note that the thermal image color-scale in this case is arbitrary.

The aerial drone was deployed either from near basecamp (51.4988367° lat., −55.6719977° lon.) or next to one of the Fat Trucks in the field to capture RGB-color and thermal images of the flooding operations (see Fig. 17). The drone was flown directly overhead at each pumping operations location to capture the shape and size of the flooded zone during and after pumping.

## Limitations

The distinction between the natural (congelation) ice and artificially flooded and thickened (snow) ice was complicated by the occurrence of significant rain events—as confirmed by timelapse camera images and thermistor chain data—which naturally flooded the snow cover and formed snow or superimposed ice. This natural process also complicated the interpretation of the boundary between the congelation ice and the snow ice due to melting and refreezing from the rain at above-freezing air temperatures. During rain events, which were followed by freezing temperatures, the lenses of the timelapse cameras were occasionally covered in a layer of ice, thereby obstructing the view. These layers of ice cleared once sunlight heated the lens.

Data collected during the melting of the ice cover might have been affected by local melting and meltwater drainage along the thermistor chain, due to additional heat conduction of the chain and wooden support structure.

The measurement station at Site B separated from the ice cover on 28 April, losing its southerly orientation and rendering the radiometer and anemometer measurements unreliable; the thermistor chain at Site B and the wooden support structure failed on 29 April. Similarly, the measurement station at Site C separated from the ice cover on 30 April and its thermistor chain failed on 4 May. The measurement station at Site A separated from the ice cover on 4 May.

The flowrate of the pumps varied considerably during operation for two reasons. First, the throttle of the pump tended towards idling when not firmly held in place, which meant that the throttle was not always full during operations. Second, the submerged depth of the impeller during installation of the pumps was not consistent due to the variability in the augering process and the snow and slush thicknesses over the ice. For these reasons, only a range of flowrates can be provided for the flood pumps.

Both ice cores and seawater samples for biological content were collected at flooded sites after flooding had occurred; no samples were collected at the reference site.

Given the relatively limited extent of the artificially thickened ice and the natural variability in sea currents, bathymetry, and temperature across the area, it is challenging to attribute differences in melting behavior primarily to the flooding intervention rather than to inherent environmental variations.

## Ethics Statement

The present study meets the ethical requirements. The authors confirm that they have read and followed the ethical requirements for publication in Data in Brief and confirm that the current work does not involve human subjects, animal experiments, or any data collected from social media platforms.

## Credit Author Statement

**Cody C. Owen**: Conceptualization, Methodology, Investigation, Data curation, Writing – Original Draft, Visualization. **Soroosh Afzali**: Conceptualization, Methodology Investigation, Data curation, Writing – Original Draft. **Willem Schellingerhout**: Methodology, Investigation, Resources. **Tom Meijeraan**: Conceptualization, Methodology, Investigation, Resources, Writing - Review & Editing, Funding acquisition, Supervision, Project administration. **Fonger Ypma**: Conceptualization, Investigation, Resources, Writing - Review & Editing, Funding acquisition, Supervision, Project administration.

## Data Availability

ZenodoField data on sea ice restoration by artificial flooding in subarctic Canada (Original data).

## Footnotes

1 ‘SIMBA_temperature_SiteA.csv’; ‘SIMBA_gps_SiteA.csv’.

2 ‘SIMBA_temperature_SiteB.csv’; ‘SIMBA_gps_SiteB.csv’.

3 ‘SIMBA_temperature_SiteC.csv’; ‘SIMBA_gps_SiteC.csv’.

4 ‘DataLogger_SiteA.csv’.

5 ‘DataLogger_SiteB.csv’.

6 ‘DataLogger_SiteC.csv’.

7 ‘Region_conditions.xlsx’.

8 ‘Snow_study.xlsx’.

9 ‘Surface_composition.xlsx’.

10 ‘Pumping_operations.xlsx’.

11 ‘Ice_core_measurements.xlsx’.

12 ‘Sea_ice_biology.xslx’.

13 ‘Timelapse_SiteA_00.mp4’.

14 ‘Timelapse_SiteB_00.mp4’; ‘Timelapse_SiteB_01.mp4’; ‘Timelapse_SiteB_02.mp4’; ‘Timelapse_SiteB_03.mp4’; ‘Timelapse_SiteB_04.mp4’; ‘Timelapse_SiteB_05.mp4’.

15 ‘Timelapse_SiteC_00.mp4’.

16 ‘Drone_images_metadata.xlsx’.

17 ‘Region_summary.mp4’.
